# Lung cancer and intraocular metastasis in gestation: Clinical experiences of a rare case

**DOI:** 10.1111/1759-7714.13572

**Published:** 2020-07-21

**Authors:** Lin Lin, Jing Sun, Jie Wang

**Affiliations:** ^1^ Department of Medical Oncology, National Cancer Center/National Clinical Research Center for Cancer/Cancer Hospital Chinese Academy of Medical Sciences and Peking Union Medical College Beijing China

**Keywords:** Cancer in gestation, choroid metastasis, lung cancer, ocular metastasis

## Abstract

Lung cancer in pregnancy combined with intraocular metastasis rarely occurs and has a poor prognosis. Here, we report an extremely rare case of a 31‐year‐old non‐smoking pregnant female who was diagnosed with lung adenocarcinoma with multiple metastasis including choroidal metastasis. Whole exon sequencing was performed but no appropriate therapeutic target was identified. The patient received standard pemetrexed plus carboplatin for first‐line treatment after delivery. Ophthalmic treatment including transpupillary thermotherapy and intravitreal ranibizumab injections were administered and the patient responded very well to treatment. Her visual acuity was restored, indicating systemic therapy combined with ophthalmic treatment was an appropriate choice of therapy.

**Key points:**

**Significant findings of the study:**

The case reported here of a pregnant female diagnosed with lung cancer with choroidal metastasis highlights the aggressive characteristics of the disease.

**What this study adds:**

Systemic therapy in combination with ocular therapy is an appropriate choice of treatment.

## Graphical abstract

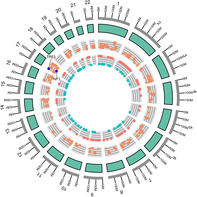

We here report an extremely rare case of a 31‐year‐old non‐smoking pregnant female who was diagnosed as lung adenocarcinoma with multiple metastasis including choroid metastasis. Whole exon sequencing was performed but no appropriate therapeutic target has been identified.

## Introduction

Lung cancer combined with intraocular metastasis and gestation is extremely rare. It has been previously recorded that only 66 pathologically‐confirmed lung cancers in gestation have been reported since 1953.^1^ The median age of patients was 36 years (17–45 years), 82% cases had non‐small cell lung cancer (NSCLC) and most were in advanced stage of disease with multiple metastases.^2^ Patients are often asymptomatic at early stages; some symptoms are confused with pregnancy manifestation and radiation screening during pregnancy is avoided resulting in a late diagnosis. Intraocular metastasis is also rare, in which the uveal tract is the most common site and occurs between 2%–9% in cancer patients, and the majority of these cases have been diagnosed with breast cancer (47%–81%) followed by lung cancer (9%–23%).^3^ Within the uvea, the choroid is the most commonly affected (88%), with retinal involvement being extremely rare.^4^ The most common symptom of intraocular metastases is blurred vision and visual loss due to macular, peripapillary retina involvement or exudative retinal detachment. In addition, about 12% of patients with ocular metastasis who have been diagnosed with lung cancer experience pain.^5^ Specialized examinations which assist in the diagnosis include optical coherence tomography (OCT), ultrasonography, magnetic resonance imaging (MRI) and computed tomography (CT). Intraocular biopsy is important for patients with no evidence of primary malignancy despite systemic investigation.

At present, little is known about lung cancer in these special circumstances. In addition, clinical treatment experiences for these patients are very limited. Previous studies have shown that the overall survival (OS) for lung cancer in gestation is 3–9 months, with 12% of patients dying within the first month post partum, and for those patients with choroid metastasis OS was six months.^6^

Herein, we present the case of a 31‐year‐old patient diagnosed with advanced lung adenocarcinoma during pregnancy who suffered visual loss as a result of ocular metastasis and subsequently received systemic treatment and ophthalmic treatment.

## Case report

**Figure 1:**
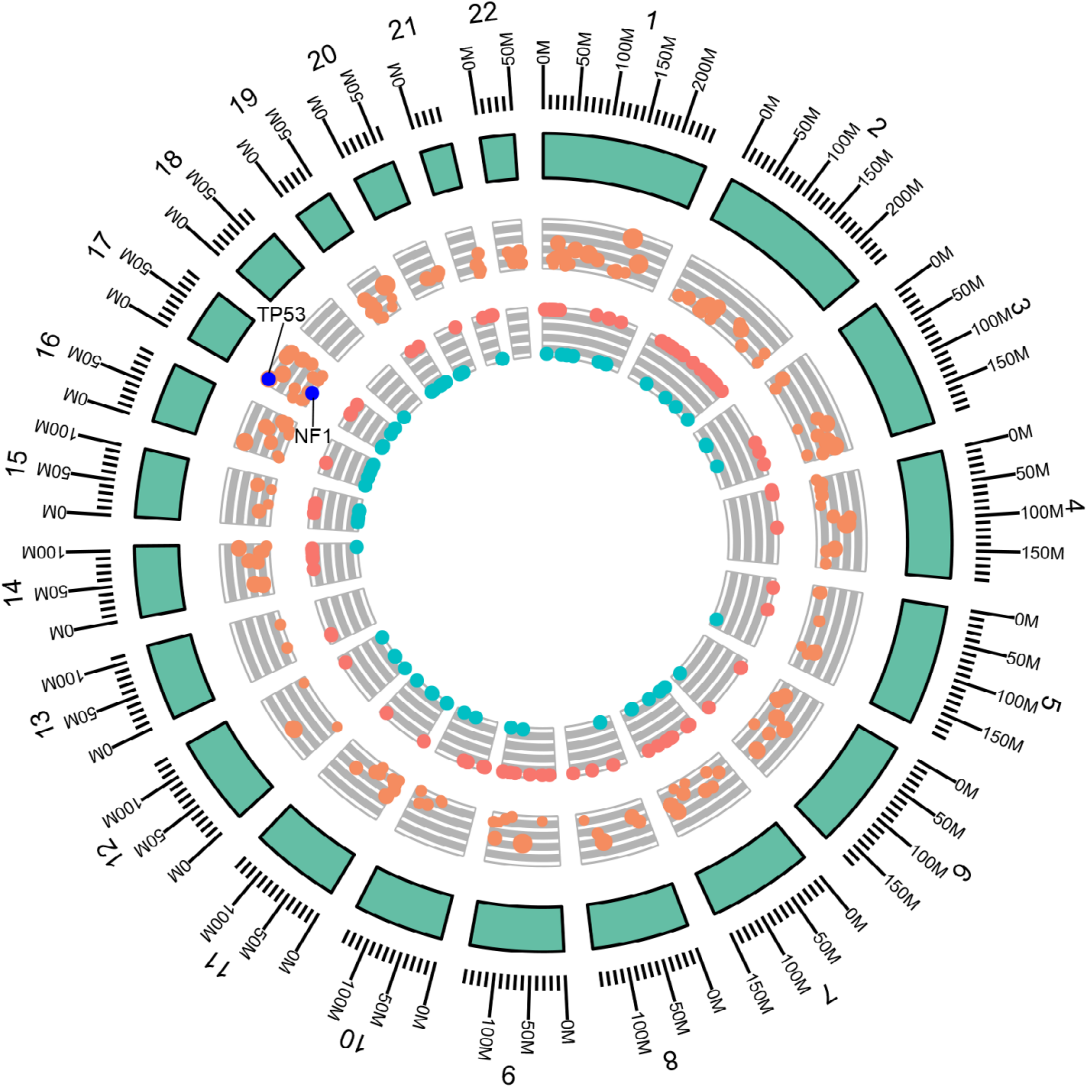
The circular plot of SNV/CNV distribution along the chromosome. The outer green circle represents the structure scale of the chromosome. The middle circle indicates the somatic variation in which its y‐axis represents the allelic fraction (AF) value of each locus. The size of points in the middle circle depict allelic depths of alternated alleles. 0 is the minimum and 1 is the maximum. The inner circle represents copy number variation (CNV). Red color indicates amplification; green color indicates deletion. Status: (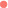) amplification, (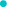) deletion; allelic_depth_alt: (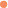) 25, (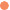) 50, (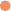) 75.

**Figure 2:**
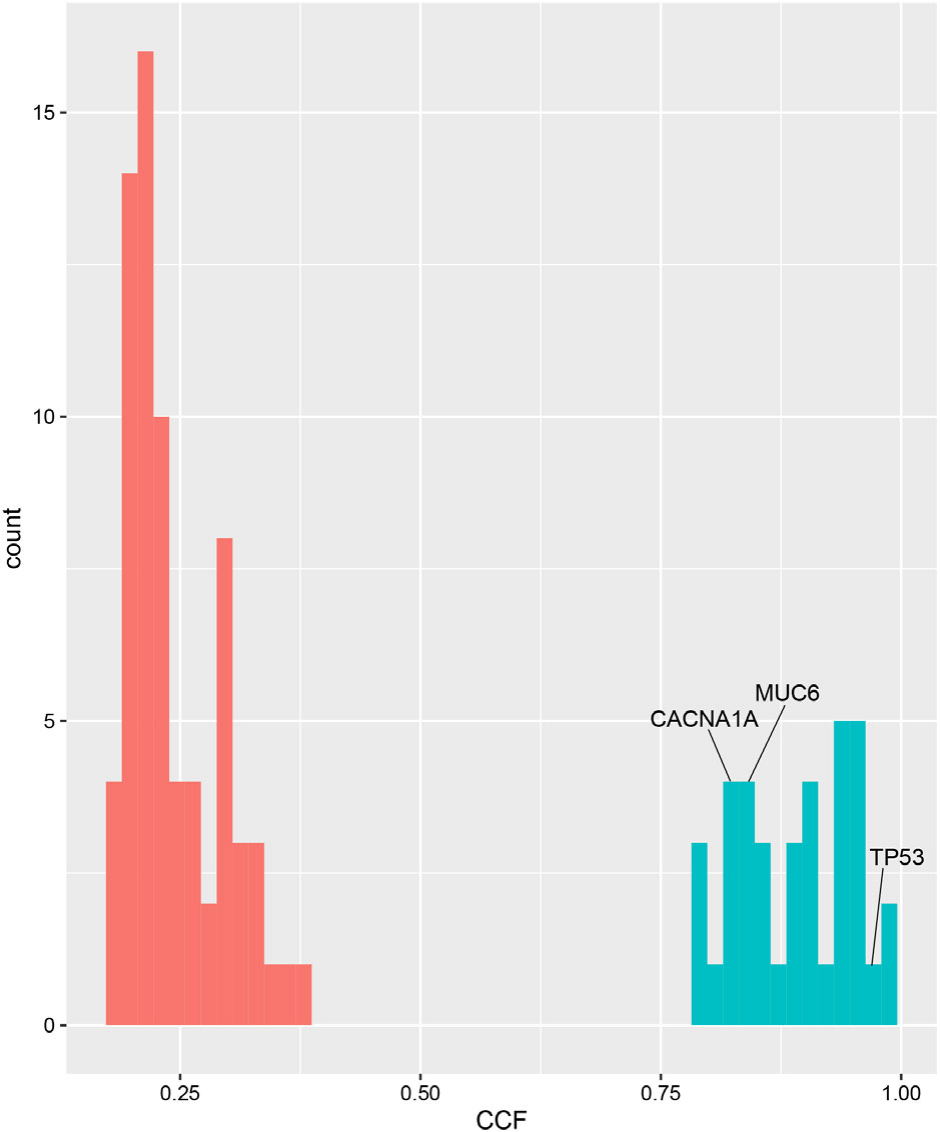
The fraction of cancer cells carrying variations in driver genes. (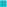) Clone; (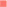) Subclone.

A 31‐year‐old female who was 26 weeks pregnant presented with intermittent cough and expectoration on December 2017. Two months later, she began to suffer right back pain. On April 2018, she developed pain in her right eye with progressive visual loss and subsequent loss of sight after delivery. Fundoscopy, OCT images and ophthalmic ultrasound identified a solid mass in the right eye, exudative retinal detachment caused by mass compression leading to visual loss, and choroidal metastasis was suspected. Further evaluation with contrast‐enhanced CT revealed a mass originating in the upper lobe of the left lung with multiple metastatic loci in the left supraclavicular (1L), mediastinum (2R, 3A, 4R/L, 5, 6, 7) and left hilar lymph nodes; and the contralateral lung, liver, left adrenal gland and bones. A lesion about 1.7 x 0.9 cm was seen in the posterior wall of the right eyeball, with moderate enhancement. Lung and mediastinal lymph node biopsy confirmed the patient had lung adenocarcinoma. Immunohistochemistry results were TTF‐1(+), c‐met (strong+>75%), ALK‐neg (−), ALK‐pos (+), ALK‐tissue (−), HER‐2 (2+), ROS‐1 (−), Ki‐67 (60%), and PD‐L1 (+5%). Whole exon sequencing (WES) of the left lung tumor tissue showed (Fig 1): *TP53*(+), *NF1*(+), tumor mutation burden (TMB) 5.9 mutations/Mb (cutoff 6.3 mutations/Mb), and MSS. We also performed clonal evolution analyses to determine the subclonal composition, and these were generated based on somatic mutations and copy number data. The fraction of cancer cells carrying variations in driver genes, *TP53*, *MUC6* and *CACNA1A*, are presented in Fig 2 and Table S1. Mutations of these three driver genes were clonal variants, and were present in high cancer cell fraction (CCF) regions.

**Figure 3:**
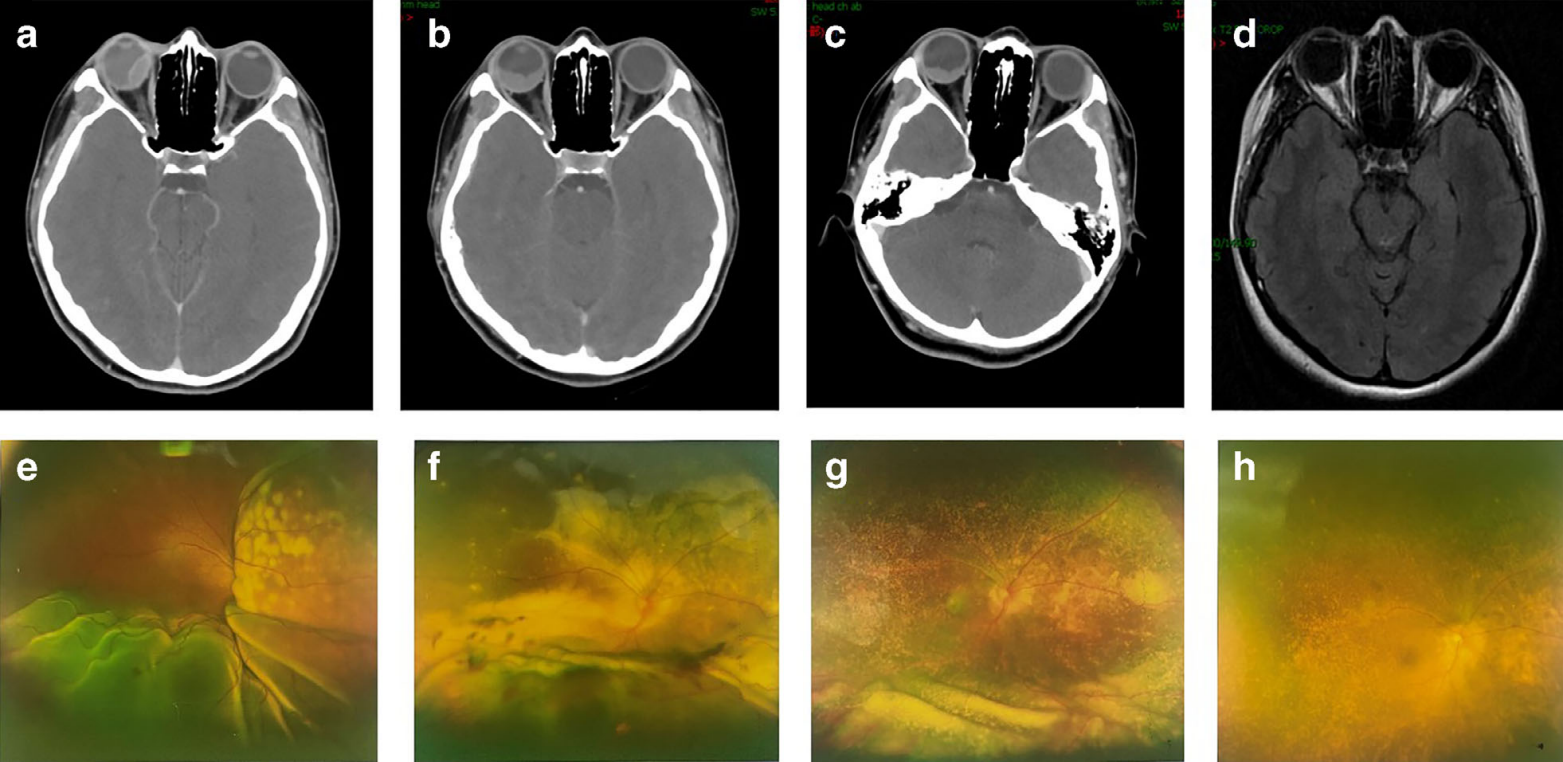
The changes in the metastatic lesion in the right eye during treatment. (**a**–**c**) Enhanced computed tomography (CT); (**d**) brain magnetic resonance imaging (MRI); (**e**–**g**) color photography. (**a**,**e**) Before all treatments; (**b,f**) after two cycles of pemetrexed/carboplatin, and transpupillary thermotherapy (TTT); (**c**,**g**) after four cycles of pemetrexed/carboplatin, TTT, one dose of ranibizumab; and (**d**,**h**) after six cycles of pemetrexed/carboplatin, and three doses of ranibizumab.

Transpupillary thermotherapy (TTT) of the right eye was performed on 25 April 2018. Systemic chemotherapy of pemetrexed plus carboplatin was administered from 14 May 2018 for six cycles (18 weeks) in total. The patient's visual acuity improved after TTT and the first cycle of chemotherapy, OCT and ultrasonography showed remission of exudative retinal detachment, especially in the macular area. The patient achieved a partial response (PR) after two cycles of chemotherapy. Ranibizumab was injected into the vitreous cavity every month, 1 mg on each occasion, three times in total. After four cycles of treatment the patient was still PR. However, the left lung lesions increased after six cycles, whereas the intraocular lesion remained stable. Second‐line chemotherapy consisting of pemetrexed plus bevacizumab was administered from 29 September 2018, and the lesions remained stable after two cycles of treatment. After three cycles, the left pulmonary lesion was found to be slightly larger than before, and radiofrequency ablation was performed. During treatment, the ocular symptoms continued to resolve, but after five cycles of treatment, the patient presented with multiple brain metastasis and pleural metastasis. The patient's condition subsequently deteriorated and she died in April 2019. The changes which occurred in the metastatic lesion in the right eye during treatment are shown in Fig 3.

## Discussion

Lung cancer during pregnancy together with intraocular metastasis is an extremely rare coincidence with a poor prognosis, and treatment for the rare case reported here was challenging. Treatment for gestational lung cancer usually begins after delivery. Use of chemotherapy during the first trimester increases the risk of spontaneous abortion, fetal death, and major fetal malformation. It has previously been reported that the combination of cisplatin and vinorelbine or etoposide have been administered in pregnant women with lung cancer without detrimental fetal effects in the second and third trimesters.^7^ Tyrosine kinase inhibitors are generally not recommended during pregnancy. A decision on pregnancy termination should be made based on the probability of cure, drugs to be used and wishes of the patient. Most pregnant patients with cancer are able to give birth to healthy babies; however, in this case maternal survival was not optimistic.

Multidisciplinary treatment is recommended for intraocular metastasis. The principle of systemic treatment is the same as that for nonocular metastasis patients. Local therapy such as radiation therapy, photodynamic therapy, TTT, and intravitreal antiangiogenic agents are also effective. However, recurrences occur in about 12% of cases. After treatment of unilateral disease, metastasis in the fellow eye is seen in approximately 15%–20% of patients.^6^

In our case, although the patient was a young, non‐smoking female, no targetable mutation was found for first‐line treatment. The low TMB and PD‐L1 indicated that the patient might not benefit from PD‐1/PD‐L1 immunotherapy. The result of WES indicated the missense mutation of *TP53* which are associated with shorter survival in patients with NSCLC.^8^ Clonal status analysis showed that there were two distinct clusters in our case, corresponding to clone and subclone, which were classified according to confidence interval of CCF. Mutations in *TP53*, *MUC6* and *CACNA1A* are supposed to be driver mutations;^9^ however, effective targeted therapies are unavailable. Therefore, standard platinum‐based chemotherapy was administered and the initial response was encouraging, especially with regard to the ocular metastasis lesions. One of the reasons for this is that the choroid is external to the blood‐retinal barrier so that systemic medications are able to diffuse freely into the metastatic lesions. Considering the significant initial effect of pemetrexed/carboplatin, pemetrexed was rechallenged combined with the VEGF inhibitor bevacizumab. The choroidal metastatic lesion was well controlled by systemic chemotherapy and local treatment including TTT and intravitreal antiangiogenetic agent ranibizumab. Progress‐free survival reached 4.5 months, and OS was 12 months which was comparable to other clinical trials.^10^

In conclusion, our case highlights the aggressive characteristics of lung cancer in gestation with ocular metastasis, and for the first time presents the WES results and clonal evolution analyses. In this study, we also demonstrated the effectiveness of pemetrexed plus carboplatin and bevacizumab in combination with local therapy for the treatment of driver mutation negative choroidal metastasis in an NSCLC patient.

## Disclosure

All authors declare that they have no conflict of interest.

## Supporting information


**Appendix S1** Supporting information.
